# Compassion fatigue in a health care worker treating COVID-19 patients: a case report

**DOI:** 10.1186/s13030-022-00239-0

**Published:** 2022-04-15

**Authors:** Tomoe Nishihara, Ayako Ohashi, Yuko Nakashima, Takafumi Yamashita, Kazutoshi Hiyama, Mika Kuroiwa

**Affiliations:** 1 Department of Psychosomatic Medicine, National Hospital Organization Fukuoka Higashi Medical Center, 1-1-1 Chidori, Fukuoka 811-3195 Koga City, Japan; 2 Department of Psychosomatic Medicine, Graduate School of Medical Sciences, Kyushu University, Fukuoka, Japan; 3 Department of Neuropsychiatry, Graduate School of Medical Sciences, Kyushu University, Fukuoka, Japan; 4 Department of Psychiatry, National Hospital Organization Fukuoka Higashi Medical Center, Koga, Japan; 5 Department of Infectious disease, National Hospital Organization Fukuoka Higashi Medical Center, Koga, Japan; 6 Department of Medicine and Biosystemic Science, Graduate School of Medical Sciences, Kyushu University, Fukuoka, Japan; 7 Department of Respiratory Medicine, National Hospital Organization Fukuoka Higashi Medical Center, Koga, Japan; 8 Department of Clinical Research Center, National Hospital Organization Fukuoka Higashi Medical Center, Koga, Japan

**Keywords:** COVID-19, Healthcare worker, Mental health, Compassion fatigue

## Abstract

**Background:**

Doctors treating COVID-19 are under extreme stress. It was reported that healthcare workers providing palliative care could present elevated levels of compassion fatigue. We herein report a case if the attending doctor of severe COVID-19 cases who felt extreme psychological difficulty and suffered from compassion fatigue.

**Case presentation:**

A 29-year-old female doctor presented with anxiety and insomnia. Her stress from overwork was exacerbated during the treatment of two related COVID-19 patients, a 47-year-old man with COVID-19 and his 76-year-old mother, who suffered acute stress disorder after the death of her son. The mother first refused treatment, but with psychiatric intervention she was able to recover and be discharged. In the course of these cases of COVID-19, their attending physician felt psychological distress and presented with insomnia and anticipatory anxiety due to the poor prognosis of the mother. After being presented with a systematic approach to improve her work situation by the hospital executive staff and undergoing psychotherapy for compassion fatigue, she recovered and was able to return to work.

**Conclusions:**

We report a physician in charge of severe cases of COVID-19, who suffered an adverse impact on her mental health. Excessively empathic engagement in the care of patients who do not survive and their relatives provides high risk for compassion fatigue. The stress-related distress of HCWs should be more widely recognized in order to improve support systems for them.

## Introduction

Coronavirus disease 2019 (COVID-19), caused by the severe acute respiratory syndrome coronavirus 2 (SARS-CoV-2), can lead to serious respiratory insufficiency and multiple organ failure through systemic inflammation [1]. Already under stress from pandemic induced factors, family members come under an even greater psychological burden, on the loss of their close relatives. Healthcare workers who feel especially strong responsibility for the treatment of their severe cases of COVID-19 may lead to excessive empathic engagement in the care of patients. They could develop compassion fatigue; “feeling the traumatic experiences of others as if they were their own” [2], as has been noted in healthcare professionals working in palliative care [3].

Herein, we report our intervention for a physician who was engaged in the inpatient care of two patients with COVID-19; a man in his 40’s and his mother who was devastated by his death. We first describe the course of the patients and later present that of their doctor in a time-oriented manner.

## Case presentation

### Patient 1

#### A 47-year-old man with severe obesity and hypertension

The patient was a well-educated, highly-skilled professional, dedicated to supporting his parents with chronic diseases. He had no psychiatric background. He developed COVID-19 after returning home from a business trip to Tokyo.

**Fig. 1:**
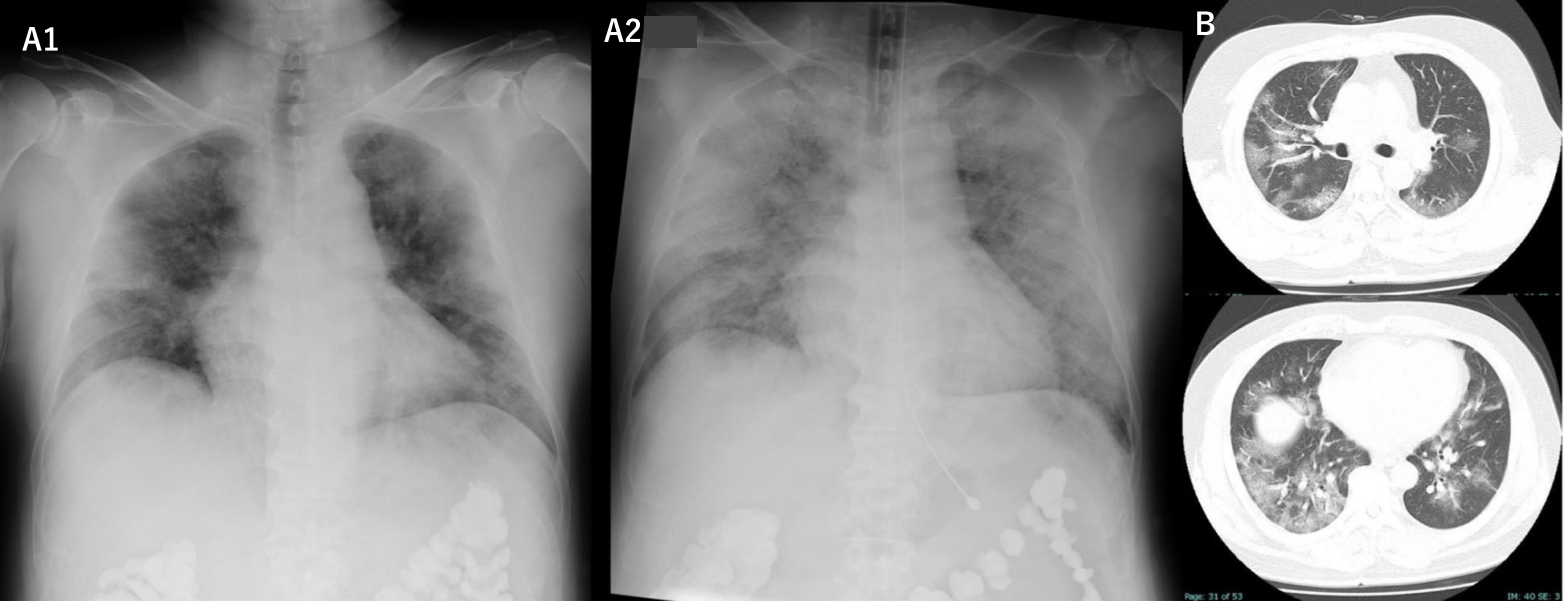
Axial images of HRCT and posteroanterior chest radiographs of patient 1. **A**, Chest radiograph obtained on hospital day2, before intubation, showed extensive interstitial shadows in both lungs (A1). Follow up radiograph after intubation revealed drastically increased opacities (A2). **B**, CT obtained on hospital day2, before intubation, showing widely spread of ground-glass opacities with crazy-paving appearance in both lungs

He presented with an eight-day history of general fatigue and later developed worsened nocturnal fatigue. He was first suspected to have infectious pneumonia because chest Radiography and HRCT showed widely spread opacities in the bilateral lung fields (Fig. 1 A1, B) and inflammatory reaction (Table 1). He was admitted to our hospital, which is designated by the Japanese Health Ministry as a center for the treatment of infectious diseases, and administered a steroid (500 mg of mPSL administered intravenously), levofloxacin hydrate (500 mg per day), and tazobactum/piperacillin hydrate. Later, because his SARS-CoV-2 RT-PCR was positive, antiviral therapy with Favipiravir (administered orally every 12 h) was started. However, his oxygenation progressively worsened, and the next day oxygen saturation was 95% with an FiO2 of 40%. With the rapid progress of respiratory failure, the patient was intubated and placed on mechanical intubation. However, his oxygenation level did not improve despite maximally intensive support with mechanical ventilation, and a massive spread of opacities was seen on chest radiography (Fig. 1 A2). He was placed on extracorporeal membrane oxygenation (ECMO), but required cardiopulmonary resuscitation to recover from recurrent cardiopulmonary arrest that occurred during ECMO. After seven hours of intensive care, he was transferred to a higher-level medical institution to continue care for multiple organ failure, but died on hospital day 11.

**Table 1:** Laboratory results

Parameter	Case 1	Case 2	Reference range
Lowest leukocyte count, ×10^3^/µl	12.3	3.9	3.3–8.6
Lowest lymphocyte, %	4.8	7	20–53
Lowest lymphocyte count, ×10^3^/µl	0.6	0.4	1-
Highest Serum ALT, U/L	62	21	10–42
Highest Serum CKP, U/L	565	269	59–248
Highest Serum LDH, U/L	548	440	124–222
Highest CRP ,mg/L	11.6	7.8	0-0.3
Highest D-dimer, mg/L	1.8	4	0-0.9
KL-6, U/mL	371	204	0-499
Ferritin, ng/ml	1751	3134	22–275
BNP, pg/ml	22.7	46.4	0-18.4

### Patient 2

#### A 78-year-old woman undergoing dialysis for chronic renal failure

**Fig. 2:**
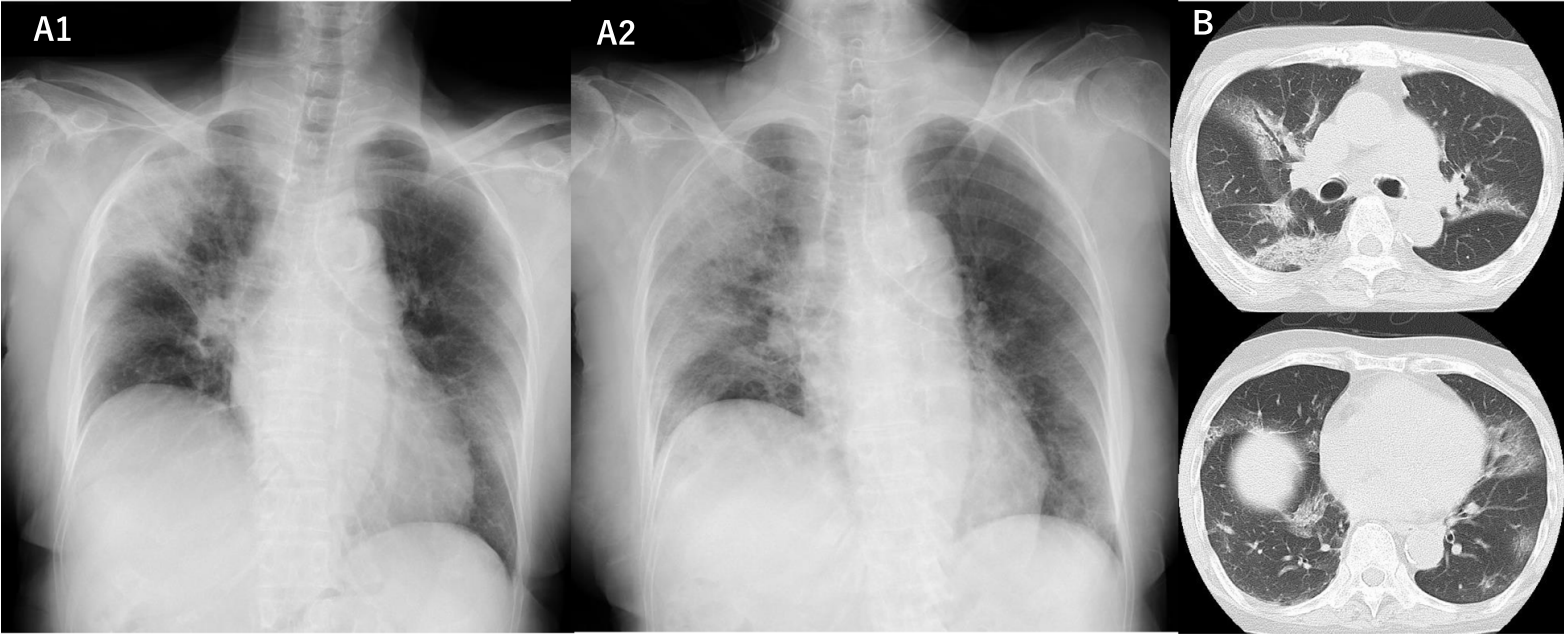
Axial images of HRCT and posteroanterior chest radiograph of patient 2. **A**, Chest radiograph obtained on hospital day 1 showed consolidation in a peripheral and mid zone distribution in right lung (A1). Follow up radiograph on hospital day 15 exhibited spread of opacities (A2). **B**, CT obtained on hospital day4, when she developed respiratory insufficiency, showing ground-glass shadow in both lungs

The patient was the mother of patient 1. She developed fever on the same day as the onset of symptoms of her son. Because her SARS-Cov-2 RT-PCR was positive, she was suspected to be infected in their family cluster and was transported to our hospital. On admission, her chest radiography showed mild shading in the right lung (Fig. 2A1), but oxygen saturation was normal. Because she lacked the findings of respiratory failure, she was followed without treatment.

However, on her second day of hospitalization, her son developed such severe respiratory failure that intensive care with ECMO was started. She was informed of his severe condition and was allowed to see him through the glass of the critical care room just before transportation. In the face of the sudden exacerbation of her son’s disease, she presented a strong stress response. She was confused and showed hyper arousal, including frequent awakenings at night. Because it was hard for her to listen to any more traumatic information, she requested not to be informed about subsequent changes in her son’s disease course. Her oxygenation level decreased over the next few days, the known shadow on her chest images was enhanced (Fig. 2 A2, B), and C-reactive protein (CRP) was elevated (Table 1). Treatment with remdesivir and dexamethasone was started. Subsequently, because her oxygen demand increased to the point that she needed oxygen flow of four to five liters per minute to maintain SpO2 > 90%, tocilizumab was administered and steroid pulse therapy was done. On hospital day 11, she was shocked and confused to hear the news of her son’s death. She was very confused and unable to express herself, with constant tearfulness and depressed facial expression. Findings of hyper arousal worsened, with night-time awakenings increased. She refused respiratory rehabilitation and medication, which he had previously been motivated to try to recover.

Her psychiatric evaluation revealed that she had acute stress disorder, presenting dissociative symptoms and increased arousal. The mental healthcare providers started psychotherapy to support her emotional recovery and medication to relieve her symptoms (5 mg of lemborexant and 4 mg of perospirone). In addition, the physicians and nurses working in the ward provided supportive communication as care for her grief. She became able to converse within a few days and decided to avoid thinking about her son, which enabled her to undergo the recommended COVID-19 treatments. She came to appreciate the consideration of the staff for allowing her to be with her son when he was in critical condition, which helped her accept the painful reality. She restarted active rehabilitation, and her respiratory failure ameliorated gradually. She was transferred to a rehabilitation hospital on hospital day 50.

### The attending physician of patients 1 and 2

#### A 29-year-old female medical doctor specializing in infectious disease

The physician was a resident physician in the Department of Infectious Diseases. She had been working as a lead person in the COVID-19 ward in the Infectious Diseases Unit for eight months, engaged in the inpatient care of more than 120 COVID-19 patients. She had no psychiatric history and no previous mental health disturbance due to the deterioration or death of her non-COVID-19 cases. Also, she did not have any personal problems outside of work. Our hospital is a designated hospital for infectious diseases, and there were two infectious diseases doctors. Her supervisor was an infectious disease specialist doctor, however, he was also an executive and focused mainly on administrative tasks. She was the only doctor in the Infectious Diseases Department to focus on clinical work on the wards. To provide medical service to the ever-increasing number of cases and with constrained resources, she was forced to undertake an overwhelming workload, working extra-shifts and longer than usual hours. Her workload had increased along with the increase of the COVID-19 outbreak, with overtime exceeding 100 h per month. She had responsibilities as the primary doctor of more than 10 COVID-19 patients.

On the day of the acute progression of patient 1, she worked over 20 continuous hours, including overseeing a series of transportation procedures and implementing simple ECMO. Later, when the respiratory condition of patient 2 worsened, it became overwhelmingly painful for her to see the deterioration of the patient in addition to the unbearable reality of her son’s condition. The physician felt psychological distress and consulted a mental health professional. She had no previous medical history and her physical examination was unremarkable. During consultation, she presented with anxiety, agitation, and insomnia. She was suffering from recurrent negative thoughts questioning if she could have provided better care for patient 1. She was also showing anticipatory anxiety because of the poor prognosis of patient 2. In addition to the increased intensity of her work load and overtime work, we saw that there was an extra psychological load because of her excessive empathic engagement in the care of the severe patient and his mother. She was diagnosed with adjustment disorder presenting as compassion fatigue and moral injury.

Her mental health condition was discussed with an industrial licensed physician and the executive staff, and she was advised to have a short-term leave or take medication. However, due to her strong sense of responsibility as the central person in the COVID-19 ward, she underwent only psychotherapy with a focus on active listening for compassion fatigue and learned self-care once a week for two months. After the report of her mental health deterioration, the hospital administration reassigned supplementary personnel from each department of internal medicine to the ward to increase the number of attending physicians for COVID-19. It worked to reduce the workload of the female infectious doctor, as well as facilitate consultation with each department, resulting in relief of her workload and heavy responsibility. Thereafter, she was able to continue working without taking a leave, partly because of the improvement in the disease course of patient 2.

**Fig. 3:**
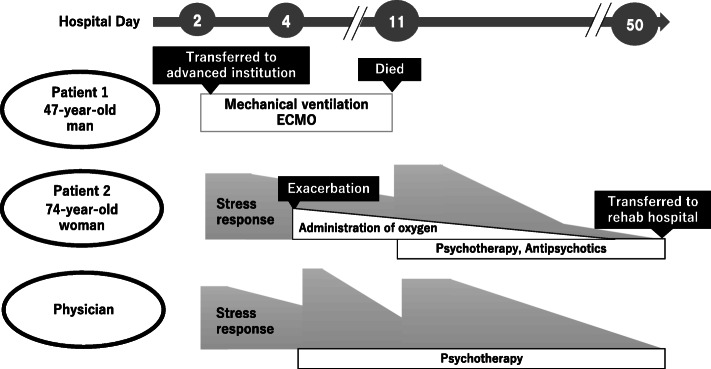
Symptoms and therapies of the patients and their physician. The stress responses of the physician closely followed the clinical course of patients 1 and 2. After experiencing the death of patient 1, she partially blamed herself. Later, when patient 2 presented exacerbation to respiratory failure, the stress reaction of the MD flared up to its maximum level, and she had anticipatory anxiety about the clinical course. When patient 1 did not survive, she showed great sympathy regarding the deterioration of the mental health of his mother. The stress of the doctor improved along with the improving course of the respiratory and psychiatric conditions of patient 2

The mental health symptoms of the physician over time are shown in Fig. 3. Her stress reaction closely accompanied the clinical course of the two patients.

## Discussion

Mental health problems such as depression, anxiety, and insomnia have been reported among frontline healthcare workers (HCWs) during the COVID-19 epidemic [4, 5]. Among the underlying psychological factors reported are fear of infection, empathic fatigue, and moral injury as representative examples [6]. However, it is difficult to assess the overall impact of moral injury or compassion fatigue because they depend on individual-level variables. The term compassion fatigue is defined as stress resulting from exposure to a traumatized individual and is described as the convergence of secondary traumatic stress and cumulative burnout [2]. To date, there have been some reports of elevated levels of compassion fatigue in mental health professionals and HCWs providing palliative care [3, 7].

Concern about moral injury, another psychological concept, was raised in the early days of the epidemic of COVID-19. Fears were raised that HCWs might have a tendency to place excessive blame on themselves and other staff because of the unprecedented and unpredictable workload [8].

In our case, the attending physician felt extreme psychological difficulty while observing the deteriorating mental and physical condition of patient 2, who had to confront not only the ultimate trauma of the sudden loss of her son, but also the exacerbation of her own physical condition. The doctor had presented self-condemnation based on the thought that she could have done more in her treatment of patient 1. Because of the combination of secondary trauma caused by empathy fatigue and excessive self-responsibility (referred to as moral injury), she was considered at imminent risk of burnout.

Our attending physician case also needs to be considered in the context of critical incident stress in disaster medicine. Critical incident stress refers to the range of emotional, mental and/or physical symptoms which cause disruption to behavior, or the ability to function either on scene or after a critical incident is over [9]. Severity of critical incident stress is affected by personal interpretation of the event, perceived seriousness of the incident, the degree of exposure, and social support from colleagues and superior or support from the organization [10]. Our female doctor case had multiple factors that are considered to be related to the severity of the incident. Her exposure to patient and family cases was significant in terms of the length of time, the extent of responsibility, and the depth of emotion because she was attending to these cases as the only their primary doctor. Prior literature has suggested that a comprehensive multifaceted approach to the management of acute stress related to a critical incident is recommended [11]. In our hospital, a systematic approach was developed and carried out by hospital executive staff to improve her work situation, including the involvement of other residents as supplemental personnel to support her clinical practice in the ward, the reinforcement of medical team by support from other departments, and the emotional support provided to the physician by the executive and clinical staffs and a mental health specialist.

There appear to be several factors that make it difficult for the personnel involved to accept the death of patients by COVID-19: (1) although there is a wide range of course, most patients recover even if they develop severe respiratory failure. Therefore, in cases resulting in death, the sudden, unexpected passing may not give sufficient time for families and caregivers to process and accept the reality. (2) the isolation from the patient necessary in the treatment of COVID-19 makes it more difficult for relatives to have a sense of reality, compared to other diseases with which they can see changes in the patient’s condition face to face. For these reasons, the HCWs in charge of the care of patients who do not survive are at risk of suffering a tremendous psychological burden because they are also responsible for the family’s mental health care.

In order to prevent the burnout of HCWs, it is important to monitor them for symptoms such as psychological distress and levels of fatigue, in the context of individual experiences. It must also be noted that stress-related physical symptoms, such as gastrointestinal symptoms, pain, and appetite loss, have been reported to be increased in frontline HCWs during the peak of the COVID-19 epidemic [10]. Mental health intervention for HCWs should include psychological education about the recognition of trauma and the need for self-care, followed by a comprehensive approach to release anxiety and reinforce self-confidence. Finally, in addition to keeping track of their workload and mental health symptoms, it is essential to share information with executives. When needed, it would be beneficial for HCWs to be provided with increased institutional support, such as by improving the medical team by enhancing manpower.

## Conclusions

We herein describe the adverse impact on the mental health of a physician in charge of a patient with a severe case of COVID-19 who died and his mother who was devastated by his loss. Deaths from COVID-19 can be traumatic not only for family members but also for the HCWs in charge of their care. Excessive empathic engagement in the care of patients who do not survive and their relatives provides high risk for compassion fatigue. These stress-related disorders of HCWs should be more widely recognized so that we can implement improved support systems for them.

## Data Availability

Not applicable.

## Abbreviations

COVID-19: Coronavirus disease 2019
ECMO: Extracorporeal membrane oxygenation
HCW: Healthcare worker
